# Profile and anticoagulation outcomes of patients on warfarin therapy in an urban hospital in Cape Town, South Africa

**DOI:** 10.4102/phcfm.v8i1.1032

**Published:** 2016-05-31

**Authors:** Babatunde O. Sonuga, Derek A. Hellenberg, Clint S. Cupido, Cilia Jaeger

**Affiliations:** 1 Division of Family Medicine, School of Public Health and Family Medicine, University of Cape Town, South Africa.; 2 Department of Medicine, Victoria Hospital, South Africa.; 3 Department of Medicine, University of Cape Town, South Africa.; 4 Department of Biotechnology, Drexel University, USA.

## Abstract

**Background:**

Warfarin is the most frequently used oral anticoagulant worldwide and it is the oral anticoagulant of choice in South Africa for reducing thrombosis-related morbidity and mortality. However, the safety and efficacy of warfarin therapy depends mainly on careful monitoring and maintenance of the international normalised ratio (INR) within an optimal therapeutic range.

**Aim:**

The aim of this study was to describe the profile and the anticoagulation outcomes of patients on warfarin therapy in a major warfarin clinic in the Western Cape Province of South Africa.

**Setting:**

Victoria Hospital - a district hospital in Cape Town.

**Methods:**

A cross sectional review of clinical records of patients on warfarin therapy who attended the INR clinic from 01 January 2014 to 30 June 2014 was done. Data analysis was done with STATA to generate appropriate descriptive data.

**Results:**

Our study showed that atrial fibrillation (AF) was the commonest indication for warfarin use in this study and hypertension was the commonest comorbidity among these patients. Only 48.5% achieved target therapeutic range; 51.5% were out-of-range. There was a significant association between alcohol consumption and poor anticoagulation outcomes (*p*-value < 0.022). Anticoagulation outcomes were better among the older age groups, male patients and in those with AF. The prevalence of thrombotic events while on warfarin treatment was 2.2%, while prevalence of haemorrhagic events was 14%. Most of the patients with bleeding events were on concurrent use of warfarin and other medications with potential drug interactions.

**Conclusion:**

In our study, patients who achieved target therapeutic control were less than the acceptable 60%.

## Introduction

Thrombosis is responsible for about 1 in every 4 deaths worldwide, and it is a significant contributor to global disease burden and mortality.^1,2,3^ Oral anticoagulant therapy (OAT) reduces morbidity and mortality associated with thrombosis-related conditions.^3^ The main treatment goal for anticoagulation therapy is to reduce the risk of thromboembolic disease in patients with atrial fibrillation (AF), mechanical heart valves, deep vein thrombosis (DVT) and pulmonary embolism (PE),^4,5^ while at the same time minimising the risk of bleeding as a result of toxicity. Available oral anticoagulants include the Vitamin K antagonists (VKAs) such as warfarin, and the newer/novel oral anticoagulants (NOACs) such as dabigatran.^6,7,8^

Warfarin is the most frequently used oral anticoagulant worldwide and it is the oral anticoagulant of choice in South Africa.^9,10,11^ Pharmacologically, the narrow therapeutic index and the highly variable toxic dose that characterizes warfarin^9,12,13^ constitute a challenge to its safe and effective use in clinical practice. Therefore, it is essential to apply best practice methods in initiation and management of patients on warfarin therapy. A Cochrane review demonstrated that warfarin is a more effective and superior oral anticoagulant than combined use of aspirin plus clopidogrel.^9,14^ The duration of anticoagulation therapy with warfarin varies from 6 months in venous thrombosis, to lifelong therapy in cardiac indications or recurrent thrombosis.^5^

Globally, management of anticoagulation therapy represents a major challenge for clinical and laboratory services.^12^ The implications of poor management of warfarin therapy are of significance to both the patient and clinician. Poor INR monitoring can result in toxicity, bleeding and increased mortality. The safety and efficacy of warfarin therapy depends mainly on careful monitoring and maintenance of the international normalised ratio (INR) within an optimal therapeutic range.^11^ The importance of therapeutic monitoring of INR is further emphasized by the fact that warfarin therapy is contra-indicated in situations when INR monitoring is not feasible.^12^

The recommended optimal or target therapeutic range for INR is 2.0–3.0 for most of the disease indications and 2.5–3.5 for those with cardiac valve prosthesis.^12,13,15,16,17^ Supra-therapeutic OAT with warfarin, with a resultant effect of high INR, puts patients at risk of warfarin toxicity or bleeding. On the other hand, sub-therapeutic anticoagulation and a sub-therapeutic INR may not protect warfarinized patients against thromboembolic disorder.^5,9,15^ Studies have shown that warfarin is greatly under-prescribed; and this has resulted in increased morbidity and mortality among affected patients.^16,17^ In 1995, a report by the Agency for Health Care Policy and Research (AHCPR) indicated that warfarin was being greatly under-utilized, because physicians are not comfortable with its safe use and fear that the drug might cause bleeding.^16^

This under-utilization of warfarin due to lack of confidence from clinicians could be interpreted as compromising patient rights to optimal care. Studies have shown that warfarin prevents 20 strokes for every bleeding episode that it causes.^16,17^ Thus, it can be deduced that the benefit of appropriate use of warfarin outweighs the risk of toxicity.^9,14^ The efforts to enhance safe warfarin therapy, aside from meticulous INR monitoring, involves patient education, good record keeping and rational drug prescription.^9,18,19,20^

There are various factors that could lead to fluctuation in the INR and also affect patient response to warfarin therapy.^11^ These factors vary from poor compliance, dosage error, concurrent illness, liver and kidney dysfunction, concomitant use of other medications, dietary interactions, laboratory error and ageing.^11,15,19^ A study done in Cape Town Metro East on comparative evaluation of warfarin utilisation at Wesfleur and Gugulethu Community Health facilities, confirmed inter-personal variability in patient response to warfarin therapy with race, gender, weight, concomitant morbidity and medications all contributing.^11^ Medications such as sodium valproate, beta-lactam antibiotics, Nonsteroidal anti-inflammatory drugs (NSAIDs) and anti-ulcer drugs appeared to alter warfarin response due to drug interactions.^11^ Vitamin K rich diets, such as kale, broccoli, cauliflower, Brussels sprouts, green tea, spinach and many green leafy vegetables also influence effectiveness of warfarin and concurrent use of some oral antibiotics with warfarin had been linked with high incidence of over-anticoagulation.^10,11,19,20,21,22,23,24^

Time in therapeutic range (TIR) is a recommended measure of outcomes of oral anticoagulation management and a good way of evaluating the quality of management of an anticoagulation clinic.^15,25^ The TIR can be calculated by 3 methods: fractions of INR in range, point prevalence (i.e. cross-section of the files), and the Rosendaal method.^20,25^ The British Committee for Standards in Haematology (BCSH) and American College of Chest Physicians Antithrombotic guidelines (which was adopted by South African Society of Thrombosis and Haemostasis) recommend that INRs should be within target therapeutic range at least 60% of time.^15,17,20^

Aside from the cost of treating warfarin adverse effects, the increasing levels of medical litigation in South Africa (and globally) is of concern to clinicians. Complications associated with over- or under-anticoagulation with warfarin could constitute a reason for litigation of health professionals. In the UK, the National Health Service (NHS) Litigation Authority has reported that anticoagulants are one of the ten most common drugs involved in errors resulting in claims against NHS trusts.^20^ However; most of these adverse effects are preventable. In South Africa, with the imminent introduction of the national health insurance (NHI) into the healthcare system, it is imperative to minimise adverse events associated with anticoagulation (warfarin) therapy by improving quality of care. There are several designated anticoagulation clinics across South Africa. A major concern however is that most of these centres do not have data on their therapeutic outcomes, the number of adverse events and bleeding incidents, in order to ensure better anticoagulation outcomes. Such records are important to positively impact decision and policy making towards optimal anticoagulation therapy. The researcher hopes that this study will improve awareness about the importance of proper oral anticoagulation and result in implementation of monitoring this service, firstly in the Western Cape and then in the rest of South Africa. Hence the motivation for the researcher to conduct a study on the profile and anticoagulation outcomes of patients on warfarin therapy in a specific centre in Cape Town.

The aim of the current study therefore was to evaluate patient profiles and the anticoagulation outcomes of patients on warfarin therapy in a major warfarin clinic in an urban hospital in Cape Town.

## Research Method

### Study design

The study was a descriptive survey that collected retrospective data from the clinical records of patients on warfarin therapy.

### Study population

The study population comprised of patients attending the INR Clinic at Victoria Hospital – a district hospital in Cape Town in the Western Cape Province, South Africa.

### Sampling method

A sample size calculation was performed based on the assumption that the percentage of patients achieving control would be at least 60%, as recommended by both the American College of Chest Physicians and the British Committee for Standards in Haematology (BCSH) guidelines on oral anticoagulation. The calculation recommended a sample size of 113 patients.

All consecutive patients who attended the INR clinic over a period of six months, between 01 Jan 2014 to 30 June 2014, and who met the study criteria were selected from the clinic attendance register. It was anticipated that this would comfortably achieve the sample size required. Patients included both old and new patients on oral anticoagulation therapy. Patients who had not been on warfarin therapy for more than 30 days were excluded from the study, because anticoagulation effects of warfarin could not be assessed yet.

### Data collection method

The folders were retrieved from the records department in October 2014 and a thorough review of the clinical record notes, treatment charts and anticoagulation record charts was conducted using excel as data extraction sheet. Parameters such as age, sex, social habits, treatment indications, existing comorbidities, INR records, warfarin use with other medications with potential drug interactions and adverse events (bleeding and thrombotic complications) were extracted for the period from 01 January to 30 June 2014. The last INR prior to 01 July 2014 for each study participant was used to categorize anticoagulation outcomes into target therapeutic range (INR 2.0–3.0 or 2.5–3.5 in patients with mechanical valve heart replacement), sub-therapeutic range (INR < 2.0 or < 2.5 in patients with mechanical heart valve replacement) and supra-therapeutic (INR > 3.0 or > 3.5 in patients with mechanical heart valve replacement).

Anticoagulation outcomes were calculated by finding the percentage of patients with last INR within target therapeutic range (% ITTR) and percentage of patients with last INR out-of therapeutic range by using cross-section-of-the-files method. This method assesses therapeutic control by taking the last INR of each patient before a pre-specified assessment date. The pre-specified assessment date for this study was 01 July, 2014. The most commonly used method of assessing anticoagulation outcome is the Rosendaal method, but it is very difficult to use in a non-computerised setting.^21^ Thus, a cross sectional method was used in this study.

### Statistical analyses

Distribution of continuous data were analysed by Shapiro-Wilk test and then the appropriate statistical methods were employed (non-parametric test, because distribution was skewed). Wilcoxon rank-sum test was used in comparing two medians, while Fisher’s exact test was used for categorical data when the expected frequency in cells was < 5.

Due to the skewed age distribution, median age and interquartile range were analysed. Differences in age and sex distribution were tested by using two sample Wilcoxon rank-sum (Mann-Whitney U) tests, while statistical association between % ITTR, age and gender distribution were tested using a Fisher’s exact test. Statistical relationship between % ITTR and various indications for warfarin, comorbidities and concurrent use of warfarin with medications with potential drug interactions were analysed with Mann-Whitney U test. Statistical association between % ITTR and bleeding or thrombotic events were tested with a Fisher’s exact test, while relationship between age and adverse events were done by using a Mann-Whitney U test. Statistical relationship between % ITTR and social habits (smoking and alcohol use) were analysed by using a Kruskal-Wallis test.

All statistical tests were two-sided. The *p*-value threshold for significance was < 0.05.

### Ethical consideration

The necessary ethics approval was obtained from the University of Cape Town Human Research Ethics Committee (HREC REF: 608/2014), Western Cape provincial research ethics committee (WC_2014RP50_937) and the management of Victoria Hospital. There was no conflict of interests and no external source of funding.

## Results

A total of 161 patients attended the clinic within the study period. Twenty three (23) patients were excluded from the study, because they were on warfarin therapy for less than 30 days. There were two missing folders, which could not be accounted for. The remaining 136 patients were all included in the study.

### Age and gender distribution of patients

Total number of patients recruited was 136; 59 males (43.4%) and 77 females (56.6%). Age range for males was between 29–85 years with median age of 62 years, while that of females was between 17–92 years with a median age of 66 years. There was a significant difference in the age distribution of patients on warfarin therapy (*p*-value < 0.029), with highest number of warfarin users (33.1%) falling between ages of 60–69 yrs in both genders (24 males and 21 females), and while the lowest number of users (6.6%) were below age 39 years. There was no statistical difference in the sex distribution among the patients who were on warfarin treatment (*p*-value < 0.179) (as shown in Table 1).

**TABLE 1:** Age and gender distribution among study population (*n* = 136).

Age distribution (years)	Female		Male		Total	
		
	Frequency	Percentage	Frequency	Percentage	Frequency	Percentage
39 and below	5	6.5	4	6.8	9	6.6
40-49	9	11.7	6	10.2	15	11.0
50-59	12	15.6	11	18.6	23	16.9
60-69	21	27.3	24	40.7	45	33.1
70-79	19	24.7	11	18.6	30	22.1
80 and above	11	14.2	3	5.1	14	10.3

**Total**	**77**	**56.6**	**59**	**43.4**	**136**	**100**

**Median ( Range)**	**66 (17-92)**	**-**	**62 (29-85)**	**-**	**-**	**-**

*Source*: Data from our study

### Social habits

#### Alcohol consumption habit

Out of the 136 patients, 88.9% (121) of patients were non-alcohol consumers, while 9.6% (13) of patients consumed alcohol and 1.5% (2) of patients had no alcohol history recorded. Out of the 13 patients who were alcohol users, four had their INR values within target therapeutic range. However, patients who consumed alcohol had lower % ITTR compared to the non-alcohol users. There was a significant association between alcohol consumption and poor anticoagulation outcomes (*p*-value < 0.022) (Table 2).

**TABLE 2:** Alcohol consumption profile among study population.

Alcohol use	Frequency among patients	Percentage	95% Confidence interval
No	121	88.9	82.4-93.3
Yes	13	9.6	5.6-15.9
Unknown	2	1.5	0.4-5.8

*Source*: Data from our study

#### Smoking habit of patients

Out of the 136 patients, 77.9% (106) of patients were non-smokers, while 19.9% (27) were smokers. Record of smoking habit was not documented in 3 (2.2%) patients. Unlike alcohol use, there was no statistical relationship between smoking habit and target therapeutic range (*p*-value = 0.198) (Table 3).

**TABLE 3:** Smoking profile.

Smoking habit	Frequency among patients	Percentage	95% Confidence interval
No	106	77.9	70.1-84.2
Yes	27	19.9	13.9-27.5
Unknown	3	2.2	0.7-6.7

*Source*: Data from our study

### Comorbidities among patients on warfarin therapy

Hypertension was the commonest comorbidity among the study population. Out of the 136 study population, 95 were hypertensive. Other common comorbidities include diabetes mellitus (37), ischaemic heart disease (35), congestive cardiac failure (34), dyslipidaemia (28) and stroke (17). Other less common comorbidities among the patients include gout (16), Chronic obstructive pulmonary disease (COPD) (14), arthritis (8), pulmonary tuberculosis (8), hypothyroidism (6), hyperthyroidism (3), chronic liver disease (2), peptic ulcer disease (1) and HIV/AIDS: positive (6), negative (15), not tested (115) (Table 4).

**TABLE 4:** Comorbidities among the patients (*n* = 136).

Comorbidities	Frequency
Hypertension	95
Diabetes mellitus	37
Congestive cardiac failure (CCF)	34
Chronic obstructive airway disease (COPD)	14
Arthritis	28
Peptic ulcer disease (PUD)	1
Tuberculosis (TB)	8
HIV	6
Liver disease	2
Gout	16
Hyperthyroidism	3
Hypothyroidism	6
Ischaemic heart disease (IHD)	35
Stroke	17
Dyslipidaemia	28

*Source*: Data from our study

### Indications for warfarin

AF was the commonest indication for warfarin use among the study population. About 65% of patients have AF as an indication for warfarin use. Other indications for warfarin use among the study population include valvular heart disease (16.9%), mechanical heart valve replacement (13.2%), DVT (13.2%), recurrent DVT (9.6%), pulmonary embolism (8.1%), hyper coagulation (2.9%) and atrial flutter (4.4%).

### Anticoagulation outcomes (cross sectional method)

**FIGURE 1:**
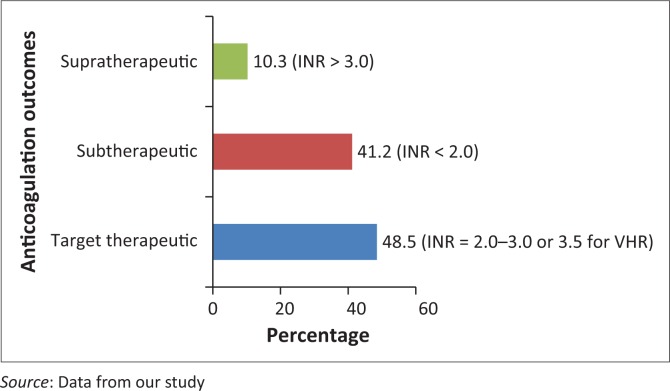
Anticoagulation outcomes of patients on warfarin.

Out of 136 patients, 66 (48.5%) had INR values within target therapeutic range as of 1 July 2014. The result showed that a total of 51.5% (70/136) of the patients were out-of-range; of which 41.2% (56) were sub-therapeutic, while 10.3% (14) were supra-therapeutic (Figure 1).

### Relationship between gender or age and anticoagulation outcomes

The study also showed that males (50.8%) have relatively higher INR within target therapeutic range than females (46.8%). The % ITTR was higher among those who are 60 years and above.

### Adverse Events while on warfarin therapy

Out of the 136 sampled population, a total of 19 patients (14%) had bleeding events (7 males and 12 females). The highest number of bleeding events occurred in both sexes among older age groups, 60 years and above. These correspond with same age group with higher % ITTR. Thrombotic events occurred in 3 patients (2.2%). The events occurred in those within age range 40-49 years (one male) and 60-69 years (a male and a female).

### Concurrent use of warfarin with other medications with potential drug interactions

A total of 87 patients were on concurrent medications with possible drug interactions with warfarin (as shown in Table 5). The most commonly used among such medications are simvastatin (57) and aspirin (35). Out of the 57 patients that were concurrently using simvastatin with warfarin, 7 reported bleeding events, while 5 patients out the 35 patients with concurrent use of warfarin with aspirin also had bleeding events. Other medications with potential drug interactions that were used concurrently with warfarin include amiodarone (7), sodium valproate (3), methotrexate (1), allopurinol (8), SSRIs (1) and digoxin (12). One of the patients on amiodarone also reported a bleeding complication.

**TABLE 5:** Indication for warfarin use in patients (*n* = 136).

Indication	Number of patients	Percentage	Number of patients with INR within target therapeutic range	ITTR (%)
Deep vein thrombosis (DVT)	18	13.2	8	44.4
Recurrent DVT	13	9.6	6	46.1
Pulmonary embolism	11	8.1	3	27.3
Heart valve disease	23	16.9	9	39.1
Mechanical heart valve replacement	18	13.2	8	44.4
Atrial fibrillation (AF)	88	64.7	46	52.3
Atrial flutter	6	4.4	2	33.3
Hyper coagulation	4	2.9	2	50.0
Cardiomyopathy/LV thrombosis	9	6.6	3	33.3

Source: Data from our study

ITTR, INR within target therapeutic range; INR, international normalised ratio.

## Discussion

Our study reported poor anticoagulation outcomes among the study population. From the result, % ITTR was 48.5%, while 51.5% of patients were out-of-range. This implied that less than half of the patients achieved optimal therapeutic outcome. A similar study in Ethiopia reported % ITTR of 30.8%, while 69.7% of patients were out-of-range.^24^ These anticoagulation reports from Africa were relatively poor outcomes when compared to a similar study in Europe, in which Poli *et al* reported a % ITTR of 71% among patients in Italy.^22^ In a study conducted across nine countries, with South Africa as one of study sites, the ACTIVE W trial gave an insight into the extent of poor anticoagulation outcomes in South Africa. The report of the trial, showed that 86% of South African patients who were entered as participants into the trial have INR that were out-of therapeutic range 60% of the time while on warfarin therapy.^14^ It was also observed in our study, that patients who were out-of-range were four times more likely to be sub-therapeutic than being over-therapeutic. In line with this finding, a similar study in Sweden showed that patients who were out-of-range were twice likely to be sub-therapeutic than over-therapeutic.^21^ But, contrary to this finding, Teklay *et al*, reported in a similar study in Ethiopia, that patients who were out-of-range were more in the supra-therapeutic range.^24^

Our study showed a statistically significant difference in age distribution of patients on warfarin therapy in our setting. The age distribution skewed more towards the older age group. Patients who were on warfarin treatment cut across different age groups. Most of the patients significantly fall between the age of 60-69 years in both males and females (*p*-value 0.029). There were more females (77) on warfarin therapy than males (59), probably because more women make use of the health facility than men. The characteristics of patients on warfarin treatment in this study population were similar in terms of age and gender distribution compared with other studies that were conducted in Cape Town and in other countries.^11,14,21,22,23,24^ Our study reported higher % ITTR among the older age groups, who were 60 years and above. This means that patients above 60 years have more INR values within target range and this implied a better anticoagulation outcome among the older age groups. A similar study in Sweden was in support of this finding and showed that there were significant correlations between time in therapeutic range and increasing age (*p* < 0.001),^23^ and that the mean dose of warfarin required decreases with advanced age, while the time spent in therapeutic range increased with age.^23^

From our study, the result also showed that male patients have better therapeutic control than the female patients (Table 6). This observation is line with the result of a similar study in Sweden, which reported that males have better anticoagulation outcomes than females.^21^ There was no sound explanation for this gender-based difference in therapeutic outcomes of patients who were on warfarin treatment. But, it could probably be as a result of consumption of more vitamin K rich diets (such as green leafy vegetables) by female patients.

**TABLE 6:** Anticoagulation outcomes.

Anticoagulation outcomes	Male		Female		Total	
		
	Number of patients	TR (%)	Number of patients	TR (%)	Number of patients	ITTR (%)
**Therapeutic category**						
Target therapeutic range	30	50.8	36	46.8	66	48.5
Sub-therapeutic	24	40.7	32	41.6	56	41.2
Supra-therapeutic	5	8.5	9	11.7	14	10.3

**Total**	**59**	**100**	**77**	**100**	**136**	**100**

*Source:* Data from our study

Fisher’s exact test: comparing relationship between gender and percentage INR within target therapeutic range (% ITTR).

*p*-value = 0.798: Time in therapeutic range and gender are not statistically related; TR, target range.

The most common indication for warfarin in our setting is AF. Similar studies conducted in the Western Cape, South Africa and in other countries in Europe and America were in agreement with this finding.^4,11,21,22,23^ AF is the most common cardiac arrhythmia worldwide.^25,26^ A systematic review of worldwide population-based studies estimated that the number of individuals with AF in 2010 was 33.5 million and that there are about 5 million new cases each year.^26^ AF increases the risk of thromboembolic stroke by 5% and warfarin treatment reduces the risk by 68%.^4,5,8,12,13^ Studies have shown that the effectiveness of warfarin in AF is reduced when INR drops below 2.0 and the effectiveness is intrinsically lost whenever INR value falls below 1.5.^15^ In our study, it was observed that patients with AF have higher % ITTR than other patients who were on warfarin for other indications as shown in Table 7.

**TABLE 7:** Adverse events.

Age interval	Males			Females			Total				
		
	Frequency	*n* (bleeding)	*n* (thrombotic)	Frequency	*n* (bleeding)	*n* (thrombotic)	Frequency	*n* (bleeding)	%	*n* (thrombotic)	%
39 or below	2	0	0	5	0	0	4	0	0.0	0	0.0
40-49	6	1	1	9	0	0	15	1	6.7	1	6.7
50-59	11	0	0	12	1	0	23	1	4.3	0	0.0
60-69	24	4	1	21	2	1	45	6	13.3	2	4.4
70-79	11	1	0	19	6	0	30	7	23.3	0	0.0
80 and above	3	1	0	11	3	0	14	4	28.6	0	0.0

**Total**	**59**	**7**	**2**	**77**	**12**	**1**	**136**	**19**	**14.0**	**3**	**2.2**

*Source*: Data from our study

This implied a relatively better anticoagulation outcome in patients who were on warfarin treatment due to AF. This finding is in agreement with the report of a similar observational study that was conducted in Italy, in which Pole *et al* described a better therapeutic control in patients with an AF than in patients with venous thromboembolism.^22^

Studies have reported that inter-individual variability and possible influence of comorbidities may affect response of patients to anticoagulation therapy.^11^ The commonest comorbidity among patients on warfarin in our study is hypertension. Chronic hypertension has been associated with complications such as AF, which has been identified as the commonest indication for warfarin therapy in our study. The role of hypertension in the epidemiology of AF is further emphasized by the fact that hypertension and valvular heart disease have been identified as the most common risk factors for AF globally.^27,28^

In our study, the effects of social habit on anticoagulation outcomes were described. The result showed a significant association between alcohol consumption and poor anticoagulation outcome (*p*-value < 0.022). Patients who consumed alcohol had lower % ITTR compared to the non-alcohol users (Table 2). Studies have shown that heavy alcohol consumption potentiates the anticoagulation effects of warfarin by increasing the INR and thereby increases the risk of bleeding.^28^ However, alcohol consumption within normal limits is safe.^28^ It is therefore important to educate patients who were taking warfarin to refrain from excessive alcohol use and for health professionals to document the quantity of alcohol consumed into the record of patients who consume alcohol. Unlike alcohol use, in our study, there was no statistical association between smoking habit and % ITTR (*p*-value = 0.198) (Table 3). In a similar study, Whitley and colleagues reported that there was no association between cigarette smoking and warfarin dose.^29^ Despite the fact that cigarette smoking has been associated with increased metabolism of several drugs, its effect on warfarin metabolism is not clearly established.^29,30,31,32^ However, smoking is an established vascular risk factor, which can independently increase the risk of thrombotic events. Almost 40% of smoking-related deaths are associated with cardiovascular disease.^30^

The Mann-Whitney U test was used to compare association between bleeding events and increasing age. Our study showed a statistically significant association between older age groups and bleeding events (*p* < 0.007). In this study, the highest number of bleeding events occurred among the older age groups above 60 years in both sexes as shown in Table 8. This finding is in agreement with other similar studies on warfarin, in which it had been reported that the incidence of both bleeding and thromboembolic events increases sharply with advanced age.^21,22,23,33^

**TABLE 8:** Concurrent warfarin use with other medications with potential drug interactions.

Concurrent drug use	Number of patients on the drug	Number of bleeding events
Amiodarone	7	1
Simvastatin	57	7
Valproate	3	0
Methotrexate	1	0
Salicylates	35	5
Allopurinol	8	0
SSRIs	1	0
NSAIDS	11	0
Digoxin	12	0

*Source*: Data from our source

NSAIDs, Nonsteroidal anti-inflammatory drugs; SSRIs, Selective serotonin reuptake inhibitors.

In our study, despite the fact that 41.2% of the INR results were sub-therapeutic, the prevalence of thrombotic events while on warfarin treatment was as low as 2.2%, while prevalence of haemorrhagic events was 14%. In a similar study, Teklay *et al* reported a haemorrhagic rate of 16.5% among patients in Ethiopia^24^, while Zhang *et al* reported a prevalence of 14.7%.^34,35^ Multivariate regression analyses of variables showed that there was a significant association between INR value and bleeding events. In our study, all the haemorrhagic events occurred when the INR values were supra-therapeutic. This finding is in support of a Norwegian study, which reported that 74% of patients who were on warfarin were supra-therapeutic at the time of bleeding event.^36^ In our setting, it was observed that, out of the 19 patients that reported bleeding events, 5 were on concurrent use of warfarin and aspirin and 7 were on concomitant use with simvastatin (Table 8). Although, this study did not assess the degree of drug interactions, a study in George sub-district in the Western Cape Province, South Africa and many other studies have reported that concurrent use of NSAIDs with aspirin increases the risk of serious bleeding.^24,34,35,37,38,39,40,41,42^ Studies have also shown that simvastatin has the potential of enhancing the effects of warfarin by inhibiting warfarin metabolism through inhibition of P450 enzymes and this might also increase the risk of bleeding.^37,38^

### Strengths and limitations

This study did not measure the actual time that each patient spent in therapeutic range. The cross sectional method used in this study only assessed a snapshot of the anticoagulation outcomes in the clinic at a specific period of time. This may not be a true reflection of what happened in the past. Also, this study did not quantify the amount of alcohol or cigarette consumed by the patients as this information was not recorded in almost all the folders that were reviewed. The advantage of the cross sectional method used in assessing the anticoagulation outcomes is that the method considers individual patients and it is not influenced by percentage of INRs out-of-range.

Future research should perhaps compare anticoagulation outcomes of patients attending primary health care based anticoagulation clinic and those attending hospital based anticoagulation clinic.

## Conclusion

In this study, patients who achieved target therapeutic control were less than the acceptable 60%. Anticoagulation outcomes were better among the older age groups and in those with AF. Bleeding complications were more common among patients on concurrent use of warfarin with other medications such as NSAIDs and simvastatin. Therefore, it is of utmost importance for health professionals to take note of drug-drug or drug-disease interactions among patients on warfarin and to monitor INR levels more frequently in patients who have to unavoidably be on concurrent use of medications with possible major interactions with warfarin.

The current South Africa anticoagulation guideline placed less emphasis on patient education. Therefore, the researcher recommend that patient education and counselling about warfarin therapy should also be given a priority during initiation of warfarin; such as it has been the standard practice before the initiation of patients on antiretroviral drugs, which had yielded a huge success in South Africa.
